# The role of plasmalemma vesicle-associated protein in pathological breakdown of blood–brain and blood–retinal barriers: potential novel therapeutic target for cerebral edema and diabetic macular edema

**DOI:** 10.1186/s12987-018-0109-2

**Published:** 2018-09-20

**Authors:** Esmeralda K. Bosma, Cornelis J. F. van Noorden, Reinier O. Schlingemann, Ingeborg Klaassen

**Affiliations:** 1 Ocular Angiogenesis Group, Departments of Ophthalmology and Medical Biology, Amsterdam Cardiovascular Sciences, Amsterdam Neuroscience, Amsterdam UMC, University of Amsterdam, Meibergdreef 9, Amsterdam, The Netherlands; 2 Department of Genetic Toxicology and Cancer Biology, National Institute of Biology, Ljubljana, Slovenia; 3 Department of Ophthalmology, University of Lausanne, Jules-Gonin Eye Hospital, Fondation Asile des Aveugles, Lausanne, Switzerland; 4 Ocular Angiogenesis Group, Department of Medical Biology, Amsterdam UMC, University of Amsterdam, Meibergdreef 15, Room L3-154, 1105 AZ Amsterdam, The Netherlands

**Keywords:** Plasmalemma vesicle-associated protein, Blood–brain barrier, Blood–retinal barrier, Cerebral edema, Diabetic macular edema

## Abstract

Breakdown of the blood–brain barrier (BBB) or inner blood–retinal barrier (BRB), induced by pathologically elevated levels of vascular endothelial growth factor (VEGF) or other mediators, can lead to vasogenic edema and significant clinical problems such as neuronal morbidity and mortality, or vision loss. Restoration of the barrier function with corticosteroids in the brain, or by blocking VEGF in the eye are currently the predominant treatment options for brain edema and diabetic macular edema, respectively. However, corticosteroids have side effects, and VEGF has important neuroprotective, vascular protective and wound healing functions, implying that long-term anti-VEGF therapy may also induce adverse effects. We postulate that targeting downstream effector proteins of VEGF and other mediators that are directly involved in the regulation of BBB and BRB integrity provide more attractive and safer treatment options for vasogenic cerebral edema and diabetic macular edema. The endothelial cell-specific protein plasmalemma vesicle-associated protein (PLVAP), a protein associated with trans-endothelial transport, emerges as candidate for this approach. PLVAP is expressed in a subset of endothelial cells throughout the body where it forms the diaphragms of caveolae, fenestrae and trans-endothelial channels. However, PLVAP expression in brain and eye barrier endothelia only occurs in pathological conditions associated with a compromised barrier function such as cancer, ischemic stroke and diabetic retinopathy. Here, we discuss the current understanding of PLVAP as a structural component of endothelial cells and regulator of vascular permeability in health and central nervous system disease. Besides providing a perspective on PLVAP identification, structure and function, and the regulatory processes involved, we also explore its potential as a novel therapeutic target for vasogenic cerebral edema and retinal macular edema.

## Background

Integrity of the blood–brain barrier (BBB) is essential to maintain a proper microenvironment for neuronal cells to function. Similar to the BBB, the inner blood–retinal barrier (BRB) protects the retina that consists of layers of specialized neurons. Breakdown of the BBB and BRB, as occurs in pathological conditions such as trauma, acute ischemic stroke [1], brain tumors [2] and diabetic retinopathy [3], causes leakage of fluid and plasma proteins from the vasculature into the surrounding tissue and subsequently vasogenic edema formation. Edema formation in the brain and eye is associated with neuronal and retinal dysfunction, causing severe mortality and loss of visual acuity, respectively [4, 5]. Given the similarities between the BBB and BRB, promising novel therapeutic candidates that prevent disruption of the BRB are also interesting targets in the treatment of BBB-related neurological diseases.

Corticosteroids are the main treatment option for brain edema, but therapy is associated with severe side effects [6]. Restoration of the barrier function via inhibition of vascular endothelial growth factor-A (VEGF), a potent inducer of angiogenesis and vascular permeability, is currently an effective treatment for diabetic macular edema (DME) and a promising treatment option for vasogenic brain edema [4, 7–10]. However, anti-VEGF therapy still has its limitations, including systemic side effects, a suboptimal response in a subset of macular edema patients, and the need for frequent and long-term therapy [11]. In addition, a large number of studies has identified roles of VEGF in wound healing and neuronal cell and retinal cell survival [12–18]. This raises significant concerns about long-term anti-VEGF therapy, indicating that alternative treatment options are needed. For such alternatives, targeting downstream effectors of VEGF, or unraveling specific cellular response pathways of barrier endothelium to VEGF, corticosteroids or other mediators, may lead to more specific and safer treatment options for vasogenic cerebral edema and DME.

Many cell types are involved in the breakdown of the BBB and BRB, but endothelial cells play a central role in this process. Under normal physiological conditions, the restrictive properties of barrier endothelial cells are the result of an extensive junctional network between cells, limited number of pinocytotic vesicles and absence of fenestrations [19, 20]. Generally, there are two main pathways by which VEGF and other mediators induce breakdown of the barrier: (1) increased paracellular transport by altering the junctional network between cells and (2) increased transcellular transport by elevated vesicular transcytosis. In the literature, the paracellular pathway is generally regarded to be the most important factor in retinal edema formation, but a growing number of studies suggest that increased transport of macromolecules via the transcellular pathway constitutes an alternative or additional mechanism of vasogenic edema formation (as reviewed in [3, 21]).

**Fig. 1:**
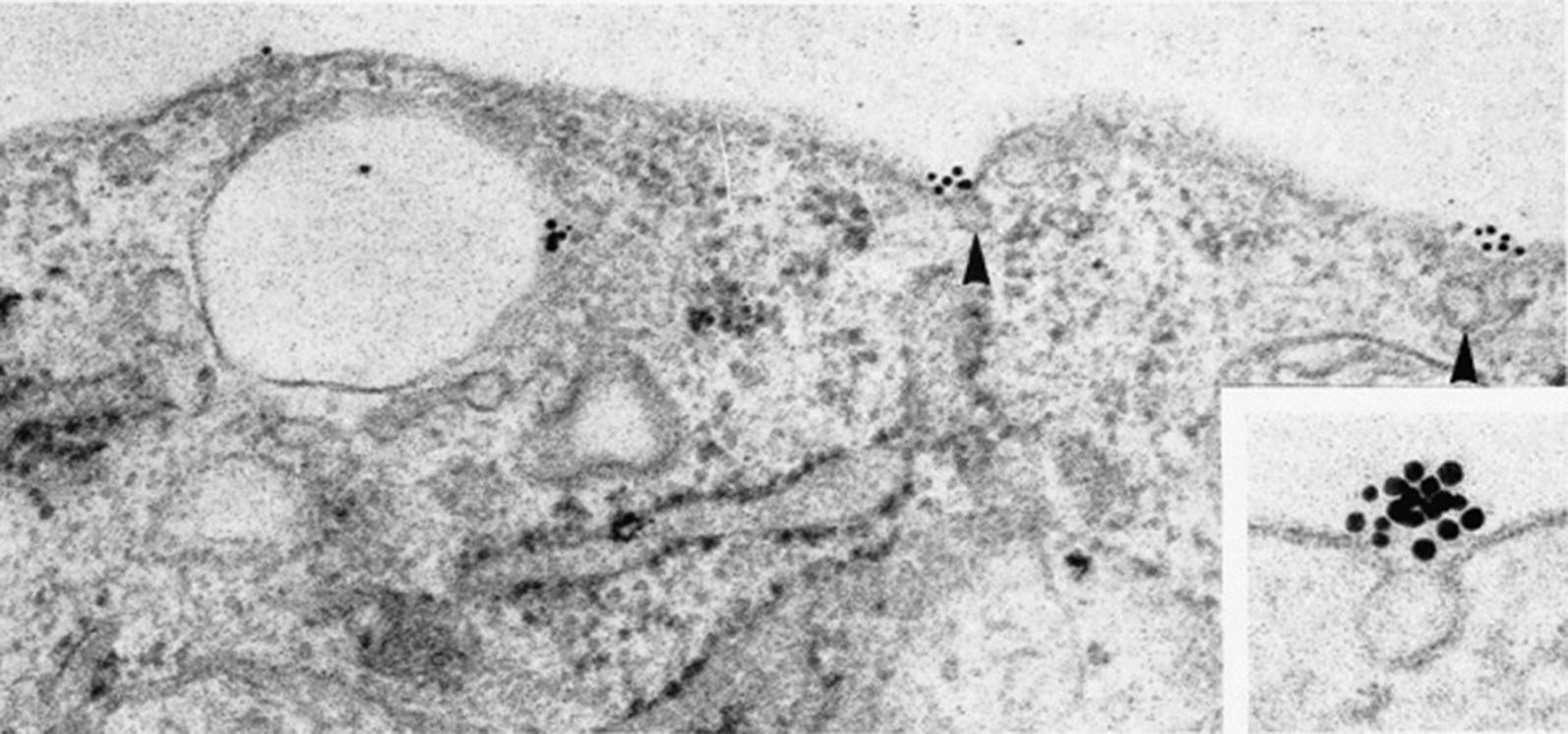
The PAL-E antibody stains endothelial vesicles. Immunogold labelling of cultured human endothelial cells from umbilical veins shows that the PAL-E antigen is associated with the exterior of endothelial vesicles (arrowheads and inset) (Reprinted from [22])

The Pathologische Anatomie Leiden-Endothelium (PAL-E) antibody, which binds an endothelial cell-specific marker expressed outside the brain and eye, has been widely used as a histochemical vascular marker in frozen tissue sections [22]. Moreover, PAL-E identifies vessels that have lost BBB or BRB properties [22–25]. Although its molecular target has remained unknown for decades, it is now well established that PAL-E recognizes plasmalemma vesicle-associated protein (PLVAP also called PV-1), a structural component of caveolae, fenestrae and trans-endothelial channels (TECs) (Fig. 1) [26, 27]. In recent years, significant progress has been made in the understanding of molecular mechanisms that are involved in the association of PLVAP and microvascular leakage. A key role of PLVAP has been elucidated in VEGF-induced BRB permeability by promoting trans-endothelial transport [28].

In the present review, we discuss the history of the identification of PLVAP, focus on the current understanding of PLVAP as regulator of vascular permeability, and explore its potential as novel target for vasogenic cerebral edema and DME.

## The history of the identification of PLVAP

The endothelium consists of a heterogeneous population of cells with diverse functions across the entire vascular system, but also within one vascular bed. Depending on different needs for controlled regulation of vascular permeability for water and solutes, the endothelium may be continuous, fenestrated or discontinuous (as reviewed in [29]). Mainly due to its specificity for endothelium and its ability to discriminate between certain subsets of endothelial cells, the PAL-E antibody has been a widely used vascular marker. PAL-E was generated over 30 years ago by injection of human melanoma lymph node metastases into mice [22]. PAL-E prominently stains endothelial cells of capillaries, medium-sized veins, venules and splenic sinusoids, but does not or only weakly stains arteries, arterioles, large veins and lymphatic vessels in tissues of mammals such as human or rabbit (Table 1) [22]. Therefore, in combination with a general vascular marker such as CD31, PAL-E is useful to distinguish between vessels of lymphatic or vascular origin. The MECA-32 antibody, which was derived from immunizing rats with murine lymph node stroma, shows similar staining patterns in mouse tissues as PAL-E in humans, and is now known to recognize the murine variant of the PAL-E antigen [30, 31]. However, it should be noted that MECA-32 but not PAL-E recognizes arterioles and large veins [22, 30].

**Table 1:** PLVAP expression in non-barrier endothelium detected with PAL-E (human tissue) and MECA-32 (mouse tissue)

	PAL-E	MECA-32	References
***Vascular system***			
Arteries			
Large elastic	–	ND	[22]
Medium-sized and small	± or −	ND	[22]
Arterioles	± or −	+	[22, 30]
Capillaries	+	±	[22, 30]
Venules	+	+	[22, 30]
Veins			
Medium-sized and small	+	ND	[22]
Large elastic	−	+	[22, 30]
***Lymphatic system***			
Lymphatic vessels	−	−	[22, 118]
Lymph node			
Subcapsular sinus	ND	+	[60]
Sinus histiocytes	−	ND	[22]
***Kidney***			
Adult kidney			
Glomeruli	−	±	[30, 119]
Endothelium^a^	+	+	[30, 119]
Fetal kidney			
Glomeruli	+	ND	[30, 119]
Endothelium^a^	+	ND	[30, 119]
***Liver***			
Adult liver			
Sinusoids	−	ND	[22]
Periportal area	+	ND	[22]
Fetal liver			
Sinusoids	+	+	[58, 98]
***Muscle***			
Cardiac muscle	±	+	[30, 33]
Skeletal muscle	±	+	[30, 33]
Bladder	+	ND	[33]
Gut	+	ND	[33]
***Other tissues***			
Lung	ND	+	[30, 55]
Pancreas	ND	+	[30, 58]
Spleen			
Sinusoids	+	+	[22, 30]
Skin	+	+^b^	[23, 55]
Thymus	+	+	[30, 45]
Intestine	ND	+	[30, 55, 58]

Grading: −, no staining; ±, variable staining; +, positive staining; ND, not determined

^a^Non-glomerular blood vessel endothelium

^b^Only reported in embryonic mouse tissue (E16.5)

In brain, PAL-E prominently stains the fenestrated endothelium of the choroid plexus, but PAL-E staining is absent in continuous endothelium of the cerebral cortex and cerebellum (Table 2) [23, 32]. Similar staining patterns are observed in the eye where PAL-E stains the fenestrated endothelium of the choriocapillaris and ciliary processes, but not the continuous barrier capillaries of the optic nerve, retina, iris and ciliary muscle (Table 2) [23, 24, 33, 34]. These findings indicate that the PAL-E antigen is absent in endothelium with BBB, BRB or other blood–tissue barrier properties. De novo expression of the PAL-E antigen in endothelium of the BBB and BRB typically occurs under pathological conditions associated with barrier disruption such as brain ischemia, cancer and diabetic retinopathy [22, 23, 25, 35–38]. Notably, expression of the PAL-E antigen in retinal vessels positively correlates with focal microvascular leakage of plasma proteins [25]. This indicates that the PAL-E antigen may actively participate in pathological BRB and BBB breakdown, underscoring the importance of resolving its molecular identity.

**Table 2:** PLVAP expression in brain and eye endothelia detected with PAL-E (human tissue) and MECA-32 (mouse tissue)

	PAL-E	MECA-32	Type of endothelium	References
***Brain***				
*Adult brain tissues*				
Parenchyma	−	−	BBB	[23, 30]
Cerebellum	−	ND	BBB	[23]
Cerebral cortex	−	ND	BBB	[23]
Medulla	−	ND	BBB	[23]
Meninges	−	ND	BBB	[23]
Midbrain	−	ND	BBB	[23]
Spinal cord	−	ND	BBB	[23]
Choroid plexus				
Lateral ventricle	+	+	Fenestrated	[23, 30]
Fourth ventricle	+	+	Fenestrated	[23, 30]
Dura mater	+	+	Fenestrated	[23, 30]
Pituitary gland	+	+	Fenestrated	[23, 30]
*Fetal brain tissues*	+	+	Immature BBB	[30, 38]
*Brain tumor tissues*				
Primary brain tumors	+	+	Disrupted BBB	[23, 35, 38]
Metastases	+	ND	Disrupted BBB	[23, 38]
***Eye***				
*Normal eye*				
Retina	−	−	BRB	[22, 24, 56, Van der Wijk et al., submitted]
Iris	−	ND	Blood–ocular barrier	[24, 33]
Optic nerve head				
Prelaminal region	+	ND	Continuous	[34]
Lamina cribosa	−	ND	BBB	[34]
Retro-laminar region	−	ND	BBB	[34]
Orbital optic nerve	−	ND	BBB	[24]
Choroid	+	+	Fenestrated	[23, 24, 56]
Ciliary process	+	+	Fenestrated	[24, 33, 56]
Ciliary muscle	−	−	Blood–ocular barrier	[24, 33, 56]
Conjunctiva	+	ND	Fenestrated	[24]
Extraocular muscle	±	ND	Continuous	[33]
Sclera	+	+	Continuous	[24, 56]
*Developing eye*				
Retina	ND	+	Immature BRB	[Van der Wijk et al., submitted]
*Diabetic eye*				
Retina	+	+	Disrupted BRB	[25, 63]

Grading: −, no staining; ±, variable staining; +, positive staining; ND, not determined; BBB, blood–brain barrier; BRB, blood–retinal barrier

Despite the fact that the molecular target of PAL-E remained an enigma for years, Schlingemann et al. [22] demonstrated in the mid-1980s that PAL-E is localized at the exterior of intracellular endothelial vesicles using immunoelectron microscopy (Fig. 1). On the basis of this specific localization and its absence in barrier endothelium, it was suggested that the PAL-E antigen is involved in trans-endothelial transport [23]. However, rather than having a universal association with endothelial vesicles, PAL-E is associated with a sub-population of vesicles that show a similar distribution as the albumin receptor in mice [23, 39].

The first study aimed at the elucidation of the molecular target of PAL-E was based on protein purification and tandem mass spectrometry analysis of tryptic peptides and identified a secreted form of vimentin as molecular target of PAL-E [40]. However, this finding was later challenged because a different antibody isotype than the original PAL-E antibody was used [22, 40]. In addition, the antibody was produced from ascites fluid which may have introduced impurities during the antibody preparation steps [27, 40]. Afterwards, Niemela et al. [27] generated the 174/2 antibody, by injection of vessels of human lymph nodes in mice and fusing the lymphocytes after immunization with myeloma cells, which recognizes PLVAP. With the use of double immunostaining, Niemela et al. [27] provided evidence that PAL-E and 174/2 recognize the same antigen and not vimentin as was reported earlier. Jaalouk et al. [41] questioned these findings since PAL-E and 174/2 did not inhibit each other in competitive staining experiments. On the other hand, they showed by epitope mapping that PAL-E recognizes a VEGF-binding site of neuropilin-1 (NRP-1) [41]. However, NRP-1 is also expressed in neuronal, epithelial and immune cells [42–44], which is in contrast to the vast number of publications that show that PAL-E and MECA-32 are endothelial cell-specific markers. The lack of competitive antigen binding in the study of Niemela et al. [27] may be explained by the notion that the different antibodies recognize different epitopes in the PLVAP molecule. While 174/2 can be used to detect PLVAP in cell lysates under reducing conditions, PAL-E only detects proteins under native conditions [27]. By performing cross-immunoprecipitations with 174/2 and PAL-E, Niemela et al. showed that PAL-E completely prevented binding of 174/2 to its molecular target (which in the control samples recognized the 120 kDa PLVAP homodimer), whereas PAL-E still recognized a protein with a molecular weight of 85 kDa after preclearing the protein lysate with 174/2 [27]. Therefore, it is plausible that PAL-E recognizes both mature *N*-glycosylated PLVAP and a degradation product, whereas 174/2 recognizes an antigenic epitope within the glycan antennae of PLVAP [27]. In conclusion, three different molecular targets of PAL-E have been proposed in the past, vimentin, PLVAP and NRP-1. However, as discussed above, the results of Niemela et al. [27] were the most convincing.

More recently, the same research group as Niemela et al. [27] provided the final evidence that PAL-E recognizes PLVAP and not NRP-1 [45]. With the use of double staining, Keuschnigg et al. [45] showed that both PLVAP and NRP-1 colocalize with the PAL-E antigen in human tissues. However, significantly different staining patterns were obtained with PAL-E and anti-NRP-1 in heart and liver, whereas the staining patterns of PAL-E and 174/2 were identical in all human tissues analyzed. To further analyze the association of PAL-E with NRP-1 and PLVAP, Keuschnigg et al. [45] transfected PLVAP and NRP-1 in HEK EBNA cells and revealed that cells transfected with PLVAP bind PAL-E and 174/2, whereas cells transfected with NRP-1 only bind anti-NRP-1. Finally, it was shown with co-immunoprecipitation studies that PLVAP and NRP-1 physically interact, explaining the contradicting findings of Jaalouk et al. [41, 45]. In an earlier study, Keuschnigg et al. [46] showed that PLVAP also physically interacts with vimentin, which may explain the findings of Xu et al. [40]. Taken together, these studies clearly demonstrated that PAL-E recognizes PLVAP.

## PLVAP structure and function

**Fig. 2:**
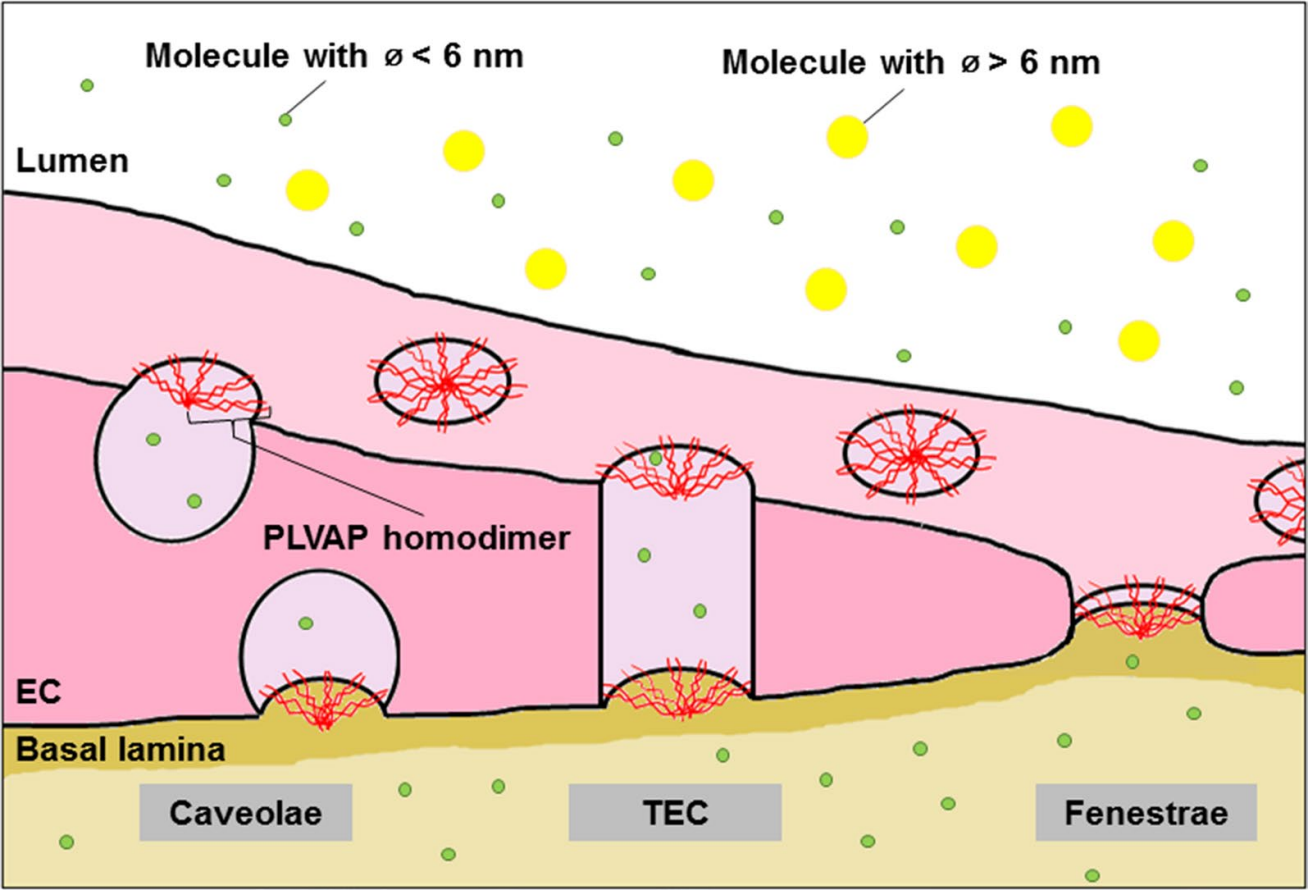
PLVAP functions as physical sieve in endothelium. PLVAP homodimers assemble in diaphragms in a wheel-like fashion. The larger part of PLVAP is extracellular, but PLVAP dimers are stabilized in the cell membrane by prominent *N*-glycosylation near its transmembrane domain (not shown) and its intracellular connections to cytoskeletal filaments (not shown). Through this distinct organization, PLVAP regulates size-dependent passage of molecules. It is hypothesized that PLVAP allows the passage of molecules smaller than 6 nm in diameter only. *EC* endothelial cell, *TEC* transendothelial channel

PLVAP is the only known molecular component of fenestral diaphragms (FDs) and stomatal diaphragms (SDs) [47, 48]. These diaphragms form cap-like structures that bridge the opening of caveolae, fenestrae and TECs (Fig. 2). Caveolae, which are characterized by the presence of caveolin-1, are spherical or flask-shaped membrane vesicles that play a role in transcytosis [49, 50]. Fenestrae and TECs are pore-like structures that allow rapid exchange of molecules between the circulation and the underlying tissue [29]. Interestingly, this nicely fits the initial observations of Schlingemann et al. that PAL-E staining is localized to the exterior of endothelial vesicles (Fig. 1), and is in line with their original hypothesis that the PAL-E antigen is involved in transcellular transport [22, 23].

PLVAP is an endothelial cell-specific type II membrane *N*-glycosylated glycoprotein with a molecular weight of approximately 55–65 kDa, that forms homodimers in situ [48, 51, 52]. PLVAP consists of a short intracellular domain (27 amino acids), a single span transmembrane domain and a large extracellular domain (380 amino acids) [48, 52]. Conserved regions are not found within the intracellular domain across species [52], but two short identical amino acids stretches of approximately 7–8 amino acids are present within the intracellular domain, of which one functions as a putative caveolin-1-binding site [49, 53]. In contrast to the intracellular domain, the extracellular domain is highly conserved in mammalian species and is characterized by four *N*-glycosylation sites, two coiled-coil domains and a proline-rich region [52]. The extracellular domain is dominated by α-helices which strongly argues for a rod-like structure [52].

The absence of PLVAP homologs in lower vertebrates that do not have SDs and FDs, and the unique expression in endothelium that contain these diaphragms support the notion that PLVAP forms SDs and FDs [49]. The precise organization and incorporation of PLVAP in SDs and FDs remains elusive. Rapid-freezing and deep-etching electron microscopy of FDs revealed that PLVAP is organized in diaphragms in an octagonal wheel-like symmetry [49, 54]. It is hypothesized that PLVAP dimers form radial fibrils that are anchored in the cell membrane via the short intracellular tail and interweaves in a central mesh via the extracellular tail [49]. The bulk of PLVAP consists of its extracellular domain, and PLVAP dimers are prevented to collapse by prominent glycosylation near the transmembrane domain which accounts for 15% of the total mass of PLVAP [48]. In addition, intracellular connections to the cytoskeleton, which occur via direct binding or cytoskeletal linker molecules [46, 49], further stabilize PLVAP in the membrane.

PLVAP expression seems to be necessary for the biogenesis of diaphragms. For instance, siRNA targeting of PLVAP prevents de novo formation of SDs and FDs after phorbol myristate acetate (PMA) stimulation, whereas transient expression of PLVAP in endothelial cells that normally lack SDs results in the formation of these diaphragms [26]. Likewise, SDs and FDs are absent in PLVAP-knockout mice [55–57]. However, other molecules are likely to be present besides PLVAP in diaphragms because fluffy electron-dense material is present in the opening of caveolae and fenestrae in PLVAP-knockout mice [55, 56]. Besides functioning as an inducer of diaphragm biogenesis, PLVAP regulates the number of fenestrae [55, 58] and caveolae in cells [28], in an VEGF-dependent manner. This indicates that PLVAP positively regulates the biogenesis of the structures in which PLVAP is expressed.

The formation of diaphragms is the only structural function of PLVAP as was concluded by Tkachenko et al. [59] on the basis of a study of two distinct vascular beds in lung and kidney in caveolin-1-knockout mice. Lung endothelium is characterized by the presence of caveolae and absence of fenestrae and TECs, whereas kidney endothelium contains caveolae, fenestrae and TECs. PLVAP protein levels were dramatically reduced in the lung vasculature but not in the kidneys of caveolin-1-knockout mice, suggesting that protein levels of PLVAP are reduced in tissues that lack the necessary structures to form diaphragms [59]. The reduced protein levels of PLVAP were not a consequence of altered transcription or translation rates because these rates were unchanged in caveolin-1-knockout mice. Rather, PLVAP appeared to be degraded in lysosomes when caveolae were absent. Therefore, it was concluded that the formation of diaphragms is the only structural function of PLVAP [59], but that does not necessarily exclude that PLVAP has other functions in endothelium.

PLVAP has a crucial functional role during embryogenesis and postnatal physiological processes. C57BL/6N or C57B1/6J PLVAP-knockout mice die before birth as a consequence of subcutaneous edema, hemorrhages, and cardiac and vascular defects [55, 57]. In a mixed C57BL/6N/FVB-N background, PLVAP-knockout mice are viable after birth and survive up to 4 weeks, but these mice are significantly smaller than their wildtype littermates, have small kidneys, spleen and pancreas, and suffer from edema, anemia and hyperlipoproteinemia [55, 58]. Similar phenotypes were observed in mice with a BALB/c–C57Bl/6J–129Sv/J background, which survive up to 3–4 months and suffer from growth retardation, edema, severe hypoproteinemia and hypertriglyceridemia [57].

Together, these results show that PLVAP plays a crucial role during the development of the cardiovascular system, and in postnatal physiological processes such as maintaining blood composition and organ homeostasis. Above all, these results indicate that PLVAP may have an important function in the regulation of vascular permeability, which will be discussed in more detail later.

Interestingly, PLVAP also plays a role in immune surveillance and inflammation. PLVAP has been shown to function as a leukocyte trafficking molecule [46]. Upon pro-inflammatory activation, PLVAP becomes redistributed in cells, allowing the formation of transendothelial channels via which leukocytes can extravasate [46]. Moreover, it was demonstrated that patches of PLVAP expression localizes together with F-actin in subcapsular sinus lymphatic endothelial cells of lymph nodes [60]. Although it is well established that PLVAP expression is absent in peripheral lymphatic endothelium, PLVAP is exclusively expressed in the lymphatic system in subcapsular sinus endothelial cells of lymph nodes, where it functions as a molecular sieve to regulate the entry of antigens and lymphocytes into the lymph node [60].

## Regulation of PLVAP expression

Several compounds, signaling molecules and biological processes have been implicated in modulating PLVAP expression, including VEGF [37, 61–64], angiotensin-2 [64], PMA [26], Norrin/Wnt-mediated β-catenin signaling [65–68], Notch signaling [69, 70], transforming growth factor-β (TGF-β) [71], inflammatory mediators such as tumor necrosis factor-α (TNF-α) [71], and shear stress [71].

VEGF, which was originally described as vascular permeability factor, is a potent inducer of both vascular permeability and angiogenesis [72, 73]. VEGF binds to three different tyrosine kinase receptors termed VEGF receptor 1–3 (VEGFR1–3). Of these receptors only VEGFR1 is highly expressed in brain and retinal vessels under physiological conditions, whereas VEGFR2 and VEGFR3 are also highly expressed in pathological conditions such as diabetic retinopathy [74, 75]. Several studies demonstrate that VEGF positively regulates PLVAP expression. For instance, VEGF but not PBS injection in monkey eyes induces PLVAP expression in retinal vessels as shown by immunohistochemistry [37]. Correspondingly, PLVAP mRNA and protein levels are increased in mice that transiently overexpress VEGF in their photoreceptors [63]. Moreover, VEGF stimulation of bovine retinal endothelial cells dramatically increased PLVAP mRNA levels [62]. With the use of selective receptor-specific engineered variants of VEGF, it was shown that PLVAP expression is upregulated in a VEGFR2-dependent manner [61]. Moreover, VEGF-dependent PLVAP expression is blocked with inhibitors of phosphatidylinositol 3-kinase (PI3K) (LY294002) or p38 mitogen-activated protein kinase (p38MAPK) (SB203580) [61]. Together, these results provide evidence that VEGFR2 signaling induces PLVAP expression in a P13K- or p38MAPK-dependent manner.

In contrast, one study showed that a VEGFR2 inhibitor increased PLVAP protein levels in lungs of caveolin-1-knockout mice, leading to the suggestion by the authors that PLVAP expression is negatively regulated by VEGF [76]. Notably, the VEGFR2 inhibitor did not alter PLVAP protein levels in wild-type mice. This rather contradictory result may be explained by the notion that VEGFR2 signaling is localized within and dependent on the caveolar compartment [77, 78]. Thus, decreased levels of caveolae in caveolin-1-knockout mice may have led to dysregulated VEGF signaling.

## PLVAP as regulator of vascular permeability

The specific tissue distribution of PAL-E antigen and the association of PLVAP with diaphragms of caveolae, fenestrae and TECs has intrigued many researchers to study its role in vascular permeability. Although PLVAP plays a crucial role in maintaining basal vascular permeability in a certain subset of endothelial cells (as described in detail below), its expression in the BBB and BRB is linked with pathological breakdown of the barrier. Here, we provide a comprehensive overview of the current knowledge regarding the role of PLVAP in vascular permeability. After a brief general introduction in vascular permeability and mechanisms of BBB and BRB breakdown, we first discuss how PLVAP modulates vascular permeability in non-barrier endothelium, before focusing on its role in the BBB and BRB and their breakdown in pathological conditions.

### Mechanism of endothelial barrier breakdown: increased vascular permeability

The formation and maintenance of a functional BBB and BRB is highly dependent on a complex interplay of multiple cell types, including endothelial cells, pericytes, astrocytes and neurons [3, 79, 80]. As such, the BBB and BRB is usually referred to as a function of the neurovascular unit. Nevertheless, endothelial cells form the direct physical barrier between the vascular lumen and the underlying tissue, and therefore their characteristic phenotype critically governs the restrictive nature of the BBB and BRB.

Endothelial cells of the BBB and BRB are characterized by the presence of an elaborate junctional network between the cells, a paucity and abluminal location of pinocytotic vesicles and absence of fenestrations [19, 20]. Under normal physiological conditions, these features of the BBB and BRB restrict the passage of potentially damaging molecules into the neuronal tissue, which is essential for proper function, in particular axonal electrical conductance [81]. However, the BBB and BRB are not impermeable barriers. There are two main routes via which molecules and solutes can cross the barrier, that is via paracellular and transcellular transport [82]. Paracellular transport occurs via the intercellular space between endothelial cells and is accomplished via dynamic regulation of the intercellular junctions such as tight junctions and adherens junctions. Transcellular transport refers to transport of molecules across endothelial cells. Transcellular transport is accomplished via transcellular diffusion, membrane transporters and vesicle-like structures termed caveolae. Outside the brain and eye, this latter type of transport occurs either via specific receptor-mediated transcytosis or non-specific fluid phase transcytosis. In the BBB and BRB, lipid-soluble molecules can passively diffuse across the barrier. In contrast, water-soluble molecules and solutes are actively transported across the barrier via receptor-mediated transcytosis via caveolae and solute transporters.

It is well established that both paracellular and transcellular transport plays a pivotal role in pathological BBB and BRB breakdown [3, 83]. Although the contribution of transcellular transport in vascular permeability has been greatly underestimated for years, this type of transport mechanism is considered to play a prominent role in edema formation. According to Starling’s rules, the net movement of fluid from capillaries is dependent on hydrostatic and osmotic pressure of the luminal and extraluminal compartments. Since macromolecules cannot easily diffuse across the membrane, we have previously hypothesized that transport of large molecules via the transcellular pathway is the main contributor to increased tissue osmotic pressure and subsequent edema formation in ocular conditions like DME [3].

VEGF has been shown to induce retinal permeability via caveolae-mediated transcytosis and not via paracellular transport [84]. In addition, intravitreal injections of VEGF in monkey eyes shifted the distribution of endothelial vesicles from an abluminal localization to a luminal localization, without clearly altering junctional integrity [85]. Moreover, caveolae are increased in numbers after VEGF stimulation of human retinal explants [28] and cultured bovine retinal endothelial cells [84]. Collectively, these findings strongly argue for a pivotal role of caveolae-mediated transcytosis in VEGF-induced BRB permeability. Although the endothelium of the BBB and BRB share highly similar characteristics, it has not been clearly established whether caveolae-mediated transcytosis also plays a central role in VEGF-induced BBB permeability. However, a recent study shows that ischemia-induced permeability in the brain is also associated with an increased number of caveolae in mouse endothelium [86], suggesting that similar mechanisms are involved.

In this review, the focus is on vasogenic edema formation which is hallmarked by a compromised barrier function of endothelial cells. However, it is important to note that neural edema can also develop as a result of aberrant ion transport while the barrier is intact. This type of edema formation, which is known as cytotoxic edema and/or ionic edema, has been extensively reviewed recently [87].

### PLVAP in non-barrier endothelium

In non-barrier endothelium, PLVAP is expressed in fenestrated endothelium (with an exception of the so-called porous endothelia in the glomerulus and liver sinusoidal capillaries) and in the continuous endothelium of skin, muscle and lung (Table 1) [22, 27, 33, 57, 88]. The development of genetic knockout mice that lack PLVAP expression has provided insight how PLVAP modulates endothelial barrier integrity in non-barrier endothelium. Analysis of the protein content of blood plasma of PLVAP-knockout mice with a mixed BALB/c–C57Bl/6J–129Sv/J background showed that total plasma protein, albumin and albumin/globulin ratios are significantly reduced with minimal electrolyte imbalance in PLVAP-knockout mice compared to control littermates [57]. This reduction in blood plasma proteins was not caused by decreased protein production, enhanced catabolism or nephropathy. In contrast, significant leakage of plasma proteins as measured with the Evans Blue dye extravasation assay was observed in organs with fenestrated capillaries (intestine, kidney, pancreas), whereas only a minimal increase of leakage in organs with continuous endothelium (heart, muscle, lung) was detected [57]. Interestingly, vascular permeability was unaltered in the liver sinusoids and glomeruli of PLVAP-knockout mice, indicating that PLVAP deletion does not alter barrier integrity in the porous endothelial cells that are known to lack FDs [57]. Collectively, these findings suggest that hypoproteinemia and hypoalbuminemia in PLVAP-knockout mice are the result of increased vascular leakage in fenestrated endothelium [57].

Recently, these results in mice were supported by two case studies of infants who had a homozygous non-sense mutation in the *PLVAP* gene, and died at 5 months and 15 days of age [89, 90]. As a consequence of this mutation, PLVAP was targeted for degradation by non-sense-mediated mRNA decay, leading to complete absence of SDs and FDs in endothelial cells [89]. Comparable to the phenotype observed in PLVAP-knockout mice, these patients suffered from severe protein-losing enteropathy which is a pathological condition characterized by excessive loss of plasma proteins in the gastrointestinal tract, and eventually death [89, 90].

Together, these observations strongly indicate that lack of PLVAP expression leads to leakage in vessels with fenestrated endothelium, in particular of proteins. Based on the localization of PLVAP in diaphragms, it is tempting to speculate that PLVAP regulates vascular permeability by providing a size limitation or filter function to fenestrae and caveolae, allowing the passage of water and solutes but preventing passage of macromolecules (Fig. 2). The ultrastructural images of Bearer et al. [54] showed that the maximum arc length between the radial fibrils is 5.46 nm on average. However, the distance between the radial fibrils may be larger than the calculated average distance since the electron microscopic technique that was used shows a larger width of the radial fibrils of 1–2 nm beyond its real size due to metal shadowing [54]. According to the assumed wheel-like incorporation of PLVAP in diaphragms with a distance between adjacent radial fibrils at the rim of approximately 6 nm [54, 60] and the physiological upper limit pore size of fenestrated endothelium and the underlying lamina basalis [57, 91], it has been postulated that lack of PLVAP fibrils in diaphragms results in vascular leakage of plasma proteins with molecular diameters between 6 and 30 nm, which includes all plasma proteins except large protein complexes or lipoprotein particles such as chylomicrons [57].

Although it has been assumed that SDs and FDs have a similar architecture and composition, several studies indicate that the different types of diaphragms have differential biochemical properties. While SDs lack anionic sites, FDs are characterized by high numbers of anionic sites [92]. This difference in charge results in an extra layer of selectivity of permeability, presumably leading to impermeability for anionic (plasma) proteins at FDs, whereas SDs may allow the passage of anionic molecules [92]. These different biochemical characteristics may be related to the glycocalyx covering the luminal side of these diaphragms, as proteases and heparinase can remove the anionic sites on FDs [93]. On the other hand, despite the fact that it is well established that the endothelial cell surface is negatively charged, the content of anionic sites on FDs appears to be considerably higher compared to that of the adjacent plasma membrane [91]. Therefore, it is tempting to speculate that PLVAP binds to different glycoproteins in different vascular beds, which may alter the chemical composition of the endothelial opening and selectivity towards molecules. However, it is also possible that other proteins besides PLVAP are present in diaphragms and this may differ in SDs and FDs.

The lack of increased permeability in the continuous endothelium of the lung of PLVAP-knockout mice in the study of Stan et al. [57], where caveolae are normally covered with SDs, made the authors conclude that the absence of PLVAP does not directly induce leakage of plasma proteins via caveolae. The apparent lack of a significant role of SDs in regulating protein extravasation is consistent with our recent observations, which show that basal vascular permeability for proteins is similar in the continuous endothelium of the dorsal skin of heterozygous *Plvap* mice as compared to wild-type mice [Van der Wijk et al., submitted]. The difference in relevance of PLVAP in SDs and FDs may be explained by the morphological differences of caveolae and fenestrae. Lack of diaphragms in fenestrae results in pores that directly connect the capillary lumen and the underlying tissue without any permselective barrier, allowing diffusion of macromolecules out of the circulation. In contrast, lack of SDs may modulate the entry of macromolecules into vesicles, but vesicular trafficking is still dependent on other proteins which include, among others, caveolin-1, dynamins and SNARE proteins that regulate caveolae formation, scission and fusion, respectively [94–96]. Alternatively, lack of FDs may considerably diminish the natural repellence against anionic proteins, which may explain why the leakage in fenestrated endothelium is so prominent. On the other hand, in vitro findings suggest that knockdown of PLVAP expression in human umbilical vein endothelial cells leads to a twofold increase in trans-endothelial electrical resistance and decreased permeability for both 70 kDa tracers and 766 Da tracers [28]. This indicates that lack of PLVAP in non-barrier endothelium may induce barrier-like properties, which is accompanied by a very low rate of vesicular transcytosis and may possibly counteract increased entry of macromolecules in vesicles when caveolae lack SDs. It is evident that PLVAP as a structural component of diaphragms has an important filter function. Therefore, we hypothesize that vesicular transcytosis in PLVAP-knockout mice is reduced to prevent aberrant transport of molecules across the barrier, and thus permeability does not increase when SDs are not present in continuous endothelium.

Besides acting as a physical sieve that controls transcytosis of molecules in mature endothelium, diaphragms may also give structural stability to caveolae and/or fenestrae during embryogenesis. In a C57BL/6N background, PLVAP-knockout mice die before birth as a consequence of subcutaneous edema and hemorrhages [55]. Transmission electron microscopy revealed that loss of SDs in subcutaneous capillaries of PLVAP-deficient embryos leads to vessels with large openings that are covered by degranulated thrombocytes [55]. This indicates that PLVAP may provide mechanical strength to the capillaries during embryogenesis, preventing vessels to become damaged during the dramatic remodeling events of angiogenesis and thus to prevent hemorrhages [55]. This function of PLVAP seems plausible as lack of FDs results in aberrant morphological phenotypes of fenestrae with widths of 50–120 nm in PLVAP-knockout mice and 20–400 nm in an in vitro assay instead of the typical 60–80 nm [57, 97], indicating that PLVAP may also give mechanical support to fenestrae. Alternatively, the lack of vessel wall integrity as observed in PLVAP-knockout mice may also be the consequence of impaired angiogenesis, as PLVAP plays an important role in this process [36].

It seems not very likely that PLVAP gives structural stability to mature endothelium, since there are also caveolae and fenestrae known to lack diaphragms in mature endothelium such as that of the kidney glomeruli and liver sinusoids [57]. PLVAP is present in these endothelia during embryogenesis [97, 98], which could be related to its function in mechanical stabilization. On the other hand, this may imply that controlled permeability is important during embryonic development.

Taken together, these results indicate that PLVAP is a crucial regulator of vascular permeability in non-barrier endothelium in both embryos and adults. During embryogenesis, PLVAP may directly promote angiogenesis or give mechanical support to capillaries which prevents the formation of leaky vessels after the dramatic remodeling steps during angiogenesis. In the mature vascular system, PLVAP has a crucial gatekeeping function, allowing the entry of small molecules but limiting leakage of large plasma proteins.

It is interesting to note that in a recent study, we found that reduced PLVAP levels in heterozygous *Plvap* mice protects the continuous endothelium of the dorsal skin from both VEGF- and histamine-induced permeability [Van der Wijk et al., submitted]. This suggests that PLVAP may function downstream of multiple permeability-inducing molecules.

### PLVAP in barrier endothelium

**Fig. 3:**
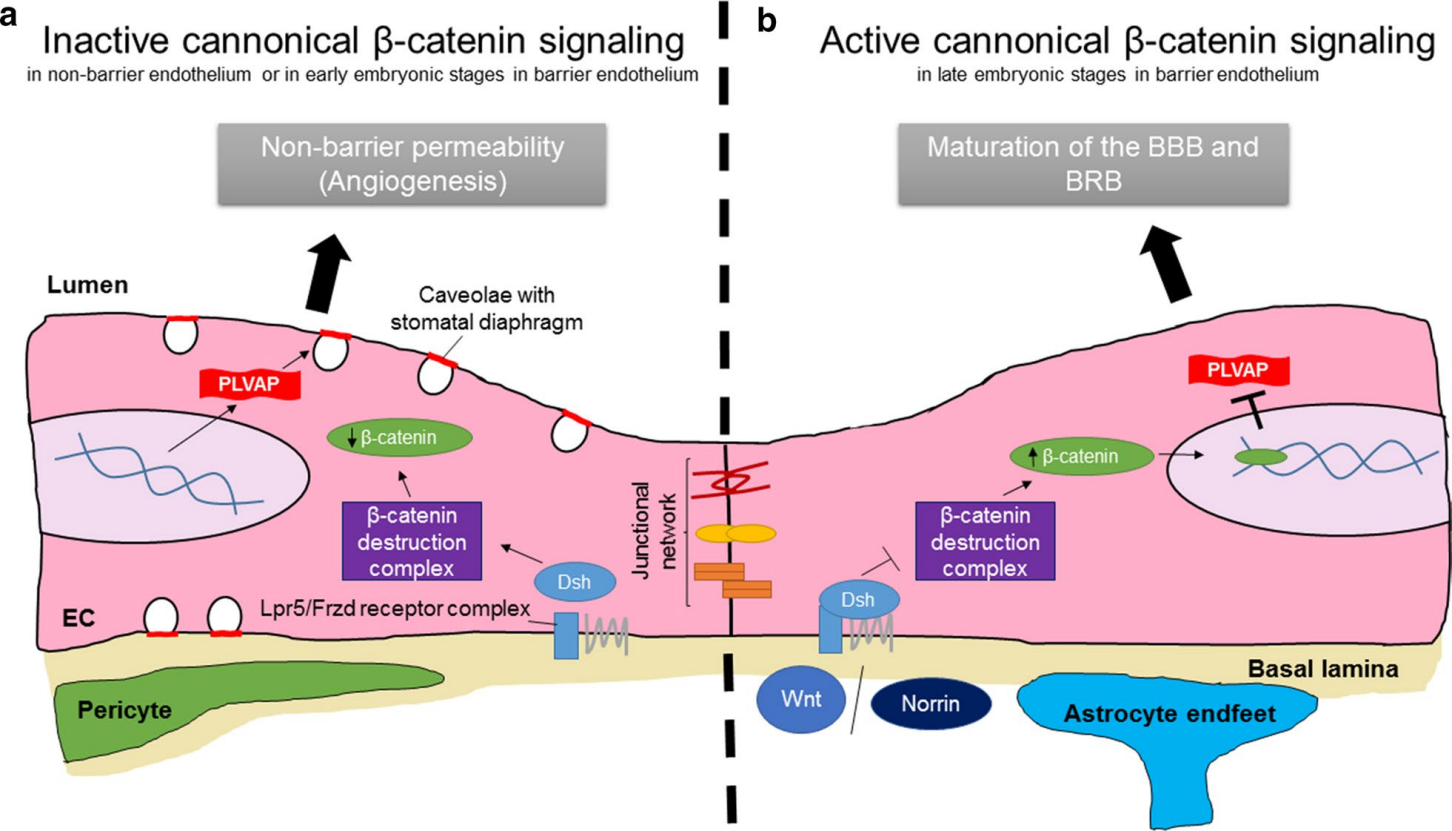
PLVAP regulates vascular development and function. **a** Absence of Wnt and Norrin ligands for the Lpr5/Frzd receptor complex results in inactive canonical β-catenin signaling in non-barrier endothelium and in early embryonic stages in barrier endothelium. As a consequence, cytosolic β-catenin is targeted for proteolytic degradation through phosphorylation by the “β-catenin destruction complex”, which consists of the APC/axin/GSK3b-complex. Low levels of β-catenin allow upregulation of PLVAP expression in endothelial cells. However, it is unknown how PLVAP expression is upregulated during vascular development. PLVAP expression in the developing vasculature is essential during angiogenesis. PLVAP may directly promote angiogenesis or give mechanical support to capillaries, which prevents the formation of leaky vessels after the drastic remodeling steps of angiogenesis. **b** The canonical β-catenin signaling pathway is active in late embryonic stages in barrier endothelium. In the presence of Wnt or Norrin ligands, the “β-catenin destruction complex” is inhibited which results in the accumulation of β-catenin in cells. Nuclear β-catenin induces the transcription of β-catenin-target genes, which results in the downregulation of PLVAP expression. Low levels of PLVAP expression induce maturation of the BRB and BBB. *EC* endothelial cell (Adapted from [68])

PLVAP expression is absent in mature endothelium with BBB and BRB properties (Table 2) [23, 24]. However, PLVAP is widely expressed in endothelia in the brain and retina during embryogenesis or postnatal development [30, 68, 99, 100, Van der Wijk et al., submitted], which is likely linked with an important function during vascular development (Fig. 3). PLVAP expression is negatively correlated with the maturation of the vasculature and acquisition of a functional BBB and BRB [30, 68, 99, 100]. Correspondingly, PLVAP expression in the BBB and BRB is induced in pathological conditions associated with vascular leakage such as diabetic retinopathy [25, 37, 75], brain ischemia [35, 36] and brain tumors [22, 23, 35]. Collectively, these observations suggest that absence of PLVAP expression in barrier endothelium is essential for the formation and maintenance of the BBB and BRB.

This is in agreement with the recent observation that suppressed transcellular transport is essential for the maturation of the BBB and BRB [101–103]. While immature and leaky retinal vessels have a functional tight junctional network that is similar to that of mature endothelium, a significant reduction in the number of vesicles governs maturation of the endothelium to form a functional BRB [101]. Further research is needed to establish the exact function of PLVAP in vascular development.

**Fig. 4:**
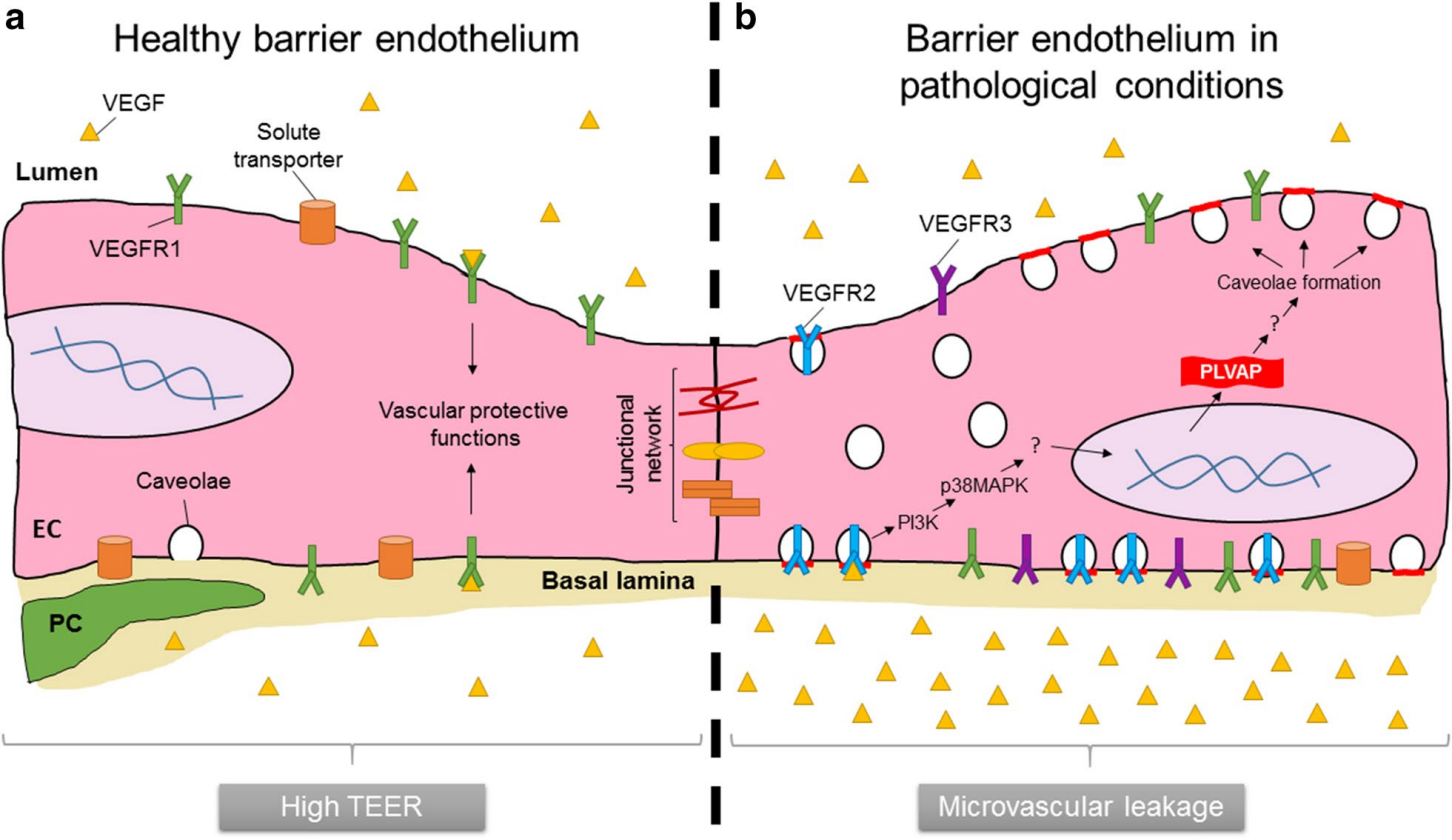
PLVAP induces vascular leakage through promoting VEGF-dependent caveolae formation. **a** The BBB and inner BRB are hallmarked by high trans-endothelial electrical resistance (TEER), which is the result of an elaborated junctional network, limited presence of caveolae and absence of fenestrae. In physiological conditions, the availability of VEGFR1 homodimers predominantly localized at the luminal side of the BBB and BRB induces important vascular protective functions [120]. In contrast, VEGFR2 homodimers at the abluminal side [120], which are probably present at a very low concentration under physiological conditions, as VEGFR2 is usually not detectable by immunohistochemistry in brain and retinal vessels [74, 75], mediate vascular leakage (not shown). **b** In pathological conditions such as diabetic retinopathy, tissue VEGF-A levels are high. In addition, VEGFR2 and VEGFR3 are highly expressed in retinal vessels of diabetic patients. Together, this results in VEGFR2/PI3K/p38MAPK-dependent PLVAP expression and formation of caveolae, which promotes leakage of plasma proteins and edema formation. *EC* endothelial cell, *PC* pericyte

In contrast, it is relatively well-established that PLVAP plays a functional role in BBB and BRB breakdown in pathological conditions (Fig. 4). With the use of short hairpin RNA (shRNA)-mediated knockdown of PLVAP expression, it was shown that PLVAP inhibition reduces VEGF-induced loss of BRB integrity as shown in a trans-endothelial electrical resistance assay [28]. Moreover, inhibition of PLVAP expression significantly reduced VEGF-induced permeability of 70 kDa tracers but not of 766 Da tracers in an in vitro model of retinal leakage [28, 104]. Correspondingly, siRNA-targeting of PLVAP expression reduced vascular permeability in the oxygen-induced retinopathy (OIR) mouse model as shown by decreased extravasation of fluorescent tracers (70 kDa) and fluorescein angiography [28]. Large molecules (70 kDa) are expected to cross the endothelial barrier via transcellular transport, whereas small molecules (766 Da) cross the barrier via the paracellular pathway. Thus, these findings suggest that (VEGF-induced) leakage via the transcellular pathway is dependent on PLVAP expression. Correspondingly, shRNA-mediated knockdown of PLVAP expression did not prevent VEGF-induced alterations in endothelial junction integrity [28]. However, stress fiber formation was significantly reduced. Ultrastructural analysis revealed that knockdown of PLVAP expression in human retinal explants blocks VEGF-induced caveolae formation to basal levels [28], suggesting that PLVAP induces leakage by enabling formation of caveolae, probably with PLVAP-containing SDs. The observation that inhibition of PLVAP expression does not modulate basal caveolae numbers is in agreement with the study of Herrnberger et al. [55], that did not observe a reduction in the number of caveolae in endothelial cells of *Plvap*-deficient mice. As previously emphasized, vesicular trafficking is a complex process that requires the need of multiple proteins and molecules [94–96], indicating that increased numbers of caveolae do not necessarily implicate increased transcytosis. However, VEGF has been shown to induce the expression of several important genes involved in the transcellular transport pathway [62] and promotes caveolae-mediated transcytosis in vivo [85], which strongly indicates that this argument is invalid during pathological vasogenic edema formation, as long as PLVAP is also expressed.

The exact molecular mechanisms by which PLVAP modulates caveolae formation or how the presence of caveolae with PLVAP-containing SDs allows barrier loss remain still unknown. Previous research indicates that VEGFR2 is localized in caveolae [77, 78]. Thus, PLVAP may modulate vascular permeability and caveolae formation via regulating the availability of VEGFR2 in cells, as a primary or secondary mechanism. Interestingly, PLVAP has been shown to interact with NRP-1, which is an important co-receptor in VEGFR2 signaling regulating VEGFR2 surface expression [45, 105]. An intriguing possibility is that PLVAP affects the stability of VEGFR2/NRP-1 complexes, and thus VEGFR2-mediated signaling. At the same time, PLVAP expression is induced in a VEGFR2-dependent manner [61]. Thus, this opens the interesting possibility of a positive feedback loop that promotes breakdown of the BBB and BRB. Further research is needed to elucidate how PLVAP regulates VEGF-dependent caveolae formation and increased permeability.

Although we have considered paracellular and transcellular transport as two independent transport mechanisms in this review so far, it is becoming clear that complex interdependency between these two pathways exist [82, 106]. In line with this insight, despite the direct association of PLVAP with transcellular transport, PLVAP appears to modulate paracellular transport as well. A direct indication that PLVAP plays a functional role in the paracellular pathway comes from the observation that PLVAP is able to regulate the number of fenestrae and caveolae in cells [28, 55, 58]. Although the exact molecular events behind the biogenesis of these structures is not known, it is evident that it requires reorganization of the cytoskeletal framework [97]. It would be interesting to determine whether PLVAP, with its intracellular connections to the cytoskeleton, affects the dynamic nature of the cytoskeleton, and subsequently intracellular gap formation. Interestingly, previous work has shown that inhibition of PLVAP expression in bovine retinal endothelial cells significantly reduced stress fiber formation after VEGF stimulation, indicating that PLVAP may regulate intracellular gap formation [28]. Although this function of PLVAP may operate via its suspected link to actin-binding proteins, it should be noted that knockdown of PLVAP expression had only limited effects on stress fiber formation in unstimulated cells [28]. This indicates that VEGF plays a central role in this process and that the role of PLVAP is indirect. A striking phenotype observed in these cells was that knockdown of PLVAP expression significantly blocked VEGF-induced caveolae formation, which led to the hypothesis that PLVAP regulates VEGFR2 availability [28]. Interestingly, VEGF-A has been shown to induce activation of RhoA, which is well known for its function in stress fiber formation and cellular contractility [107, 108]. Hence, PLVAP may modulates stress fiber formation via induction of VEGFR2-dependent Rho GTPase signaling in cells, which is an interesting avenue to be explored in future research. Moreover, in a recent study we have found that expression of tight junctions and adherens junctions is altered in the retina of heterozygous *Plvap* mice [Van der Wijk et al., submitted]. Although it is not known how this altered expression is translated into functional junctional characteristics, it indicates that PLVAP affects paracellular transport as well.

In short, PLVAP plays an important functional role in VEGF-induced BBB and BRB breakdown by regulating the number of caveolae with PLVAP-containing SDs. Although the sieving function of PLVAP during macromolecular transport in fenestrated endothelium is well established, PLVAP regulates vascular permeability in multiple and complex additional ways that remain incompletely understood.

## Therapeutic perspective for cerebral edema and diabetic macular edema

Treatment approaches for vasogenic cerebral edema and DME remain limited. Available options for cerebral edema include osmotherapy, corticosteroids and surgery [109]. However, osmotherapy only has transient effects, and its overall effectiveness remains elusive [110]. In contrast, corticosteroids are well-known to reduce cerebral edema, but therapy is associated with numerous severe side effects that may persist after treatment [6]. For this reason, the identification of novel therapeutic targets for cerebral edema is of high clinical importance. Several studies demonstrate that VEGF inhibition reduces cerebral edema that is caused by ischemic stroke and brain tumors [4, 8, 9]. However, VEGF is an important signaling molecule known to regulate pleiotropic cellular responses including neuronal survival and function [12, 14, 18], indicating that reduction of VEGF levels may have significant clinical consequences. In addition, systemic anti-VEGF therapy in cancer patients is associated with a high risk of stroke and other arterial thrombo-embolic events [111, 112].

Apart from the management of risk factors such as hyperglycemia and hypertension [113], current treatment options for DME consist of anti-VEGF agents, corticosteroids and laser photocoagulation. Laser photocoagulation has remained the standard care of treatment for over 30 years, but currently anti-VEGF therapy is the first line of treatment for DME [11]. However, agents that block VEGF do not target the initial upstream factors that lead to a compromised barrier which makes repeated injections (every 4–6 weeks) necessary [17]. In addition, a tight balance between VEGF and the pro-fibrotic growth factor termed connective tissue growth factor (CTGF) is present in the eye [15, 114]. Anti-VEGF therapy in patients with DME accompanied by severe proliferative DR has been shown to cause a shift in balance of these growth factors, favoring CTGF levels, and causing retinal fibrosis [15]. Recently, it was shown that repeated intravitreal anti-VEGF injections induces retinal neurodegeneration and increased vascular leakage in Akita mice (Ins2^Akita^) [17]. Thus, this suggests that long-term anti-VEGF therapy induces retinal damage, which may eventually have significant impact on visual function. Although the effects of long-term anti-VEGF therapy in the brain remain unknown, the same characteristics of brain and retinal barrier endothelium and the observed neurodegenerative effects in the study of Hombrebueno et al. [17] indicate that long-term therapy may also induce serious side effects in the brain.

In contrast to VEGF which has effects on multiple cell types, PLVAP is only expressed in endothelial cells, where it modulates caveolae formation and possibly VEGFR2 availability [28]. Consequently, targeting PLVAP expression in brain and retinal endothelial cells may inhibit downstream responses of VEGF and of other mediators in endothelial cells without altering cell survival of neurons [12, 14, 18]. Together with the striking observation that PLVAP inhibition significantly reduced vascular leakage of large macromolecules in vitro and in vivo [28], it strongly indicates that anti-PLVAP therapy may hold great potential as a safer novel treatment option for vasogenic cerebral edema and DME.

The widespread expression of PLVAP in non-barrier continuous endothelia and the crucial functions of PLVAP as a regulator of vascular homeostasis in fenestrated endothelium indicate that systemic targeting of PLVAP is not recommended. However, the ease of local drug delivery in the eye, and the limited fluid and molecular exchange between the eye and the rest of the body indicate that local PLVAP inhibition is achievable. For instance, PLVAP expression can be inhibited via intravitreal injections and slow-release drug delivery systems such as implantable micropumps [115] or biodegradable polyesteramide microspheres [116]. In contrast to the eye, local drug delivery to the brain may be more problematic. However, in the case of tumor-related cerebral edema, which often requires surgery to remove as much as possible tumor tissue, anti-PLVAP therapy may be implemented during surgery to restore the functional properties of the BBB to prevent relapse of edema formation.

Further investigations of the therapeutic rationale and possibilities of anti-PLVAP therapy is warranted. Interestingly, PLVAP has been shown to modulate angiogenesis as well [36], which indicates that PLVAP is also a target for conditions associated with angiogenesis such as age-related macular degeneration, proliferative diabetic retinopathy and cancer.

## Therapeutic perspective: targeted drug delivery via caveolae

Besides the potential to restore the function of barrier endothelia via inhibition of PLVAP expression, PLVAP may also be exploited to achieve targeted drug delivery across the BBB and BRB in pathological conditions. In a recent study, the therapeutic enzyme superoxide dismutase (SOD) was conjugated to antibodies against PLVAP with the ultimate goal to target SOD to caveolae of endothelial cells of pulmonary vessels, as the target substrate of the enzyme is present in endothelial vesicles [117]. Interestingly, SOD conjugated to PLVAP antibodies more efficiently blocked lipopolysaccharide-induced pulmonary inflammation than SOD conjugated to endothelial cells via CD31 [117]. It is interesting to assess whether such type of drug delivery has potential for the BBB and BRB.

## Concluding remarks

PLVAP is an important regulator of vascular permeability during embryogenesis, and after birth in fenestrated endothelium and in pathological conditions, in particular of macromolecules. Although the sieving regulatory function of PLVAP as a molecular component of diaphragms remains comprehensible, it remains incompletely understood how PLVAP modulates breakdown of the BBB and BRB. Previous research indicates that PLVAP induces vascular permeability via promoting VEGF-dependent caveolae formation, but the molecular mechanisms remain unclear [28]. Furthermore, very little is known how VEGF induces PLVAP expression. There are indications that this occurs via a VEGFR2/PI3K/p38MAPK-dependent signaling pathway [61], but it remains unknown which transcriptional regulators are involved in this process. More research is needed to better understand the underlying molecular and cellular mechanisms that link PLVAP to vascular leakage and breakdown of the BBB and BRB. Nevertheless, after decades of functioning as histological marker for endothelium in normal tissues outside the brain and eye,  and in pathological leakage in the brain and eye without a known molecular substrate, the first functional evidence has now been provided that PLVAP plays a pivotal role in VEGF-induced BRB permeability. Together with the selective expression of PLVAP in the BBB and BRB during pathological conditions, PLVAP emerges as a novel promising therapeutic target to prevent the clinical burden of vasogenic cerebral edema and DME.

## Abbreviations

BBB: blood–brain barrier
BRB: blood–retinal barrier
CTGF: connective tissue growth factor
DME: diabetic macular edema
FD: fenestral diaphragm
NRP-1: neuropilin-1
OIR: oxygen-induced retinopathy
p38MAPK: p38 mitogen-activated protein kinase
PAL-E: Pathologische Anatomie Leiden-Endothelium
PI3K: phosphatidylinositol 3-kinase
PLVAP: plasmalemma vesicle-associated protein
PMA: phorbol myristate acetate
PV-1: plasmalemma vesicle-associated protein
SD: stomatal diaphragm
shRNA: short hairpin RNA
SOD: superoxide dismutase
TEC: trans-endothelial channel
TGF-β: transforming growth factor beta
TNF-α: tumor necrosis factor alpha
VEGF: vascular endothelial growth factor
VEGFR: vascular endothelial growth factor receptor
